# STIRRING THE POT OF AGING: DIVERSITY IS THE SECRET INGREDIENT

**DOI:** 10.1093/geroni/igae098.1401

**Published:** 2024-12-31

**Authors:** Alisha Thompson, Barbara Mendez Campos, Laneshia Conner

**Affiliations:** Chair; Co-Chair; Discussant

## Abstract

Aging and our experiences of this process are as unique as the ingredients in our favorite recipes that warm our hearts on chilly days. These components of diversity are crucial factors that influence our health outcomes and well-being across our lifespan, especially as we age. Empirical evidence highlights the importance of understanding how these factors flavor our experiences of aging. Through a series of presentations and discussions, we will explore factors often thought of when considering diversity such as race and ethnicity. Discussed will be how these factors influence end-of-life care preferences when contending with a serious illness and our ethical responsibilities to deliver culturally sensitive care. Next will be a discussion of the often-overlooked issue of intimate partner violence (IPV) among older immigrants. Emphasized will be IPV prevalence and its nuanced presentation among older immigrants. We will then expand our understanding of diversity, beyond race and ethnicity, by looking at additional factors of diversity such as differing cognitive, hearing, and visual abilities. The connections between sensory impairments and pain experiences and disparities in telehealth usage among patients with hearing, visual, or cognitive impairments will be elucidated. This symposium offers a comprehensive perspective on navigating the challenges faced by our diverse aging population. By fostering dialogue on these critical topics, we aim to empower healthcare providers, policymakers, and advocates to create a more flavorful and equitable future for older adults. Join us in stirring the pot of aging as we highlight that diversity is the unique secret ingredient.

